# Prevalence and burden of orthopaedic implantable-device infections in Italy: a hospital-based national study

**DOI:** 10.1186/s12879-020-05065-9

**Published:** 2020-05-12

**Authors:** Luca Pirisi, Federico Pennestrì, Marco Viganò, Giuseppe Banfi

**Affiliations:** 1 Confindustria Dispositivi Medici, Via Burigozzo 1, 20122 Milan, Italy; 2 IRCCS Orthopedic Institute Galeazzi, Via Riccardo Galeazzi 4, 20161 Milan, Italy; 3 Vita-Salute San Raffaele University, Via Olgettina 58, 20132 Milan, Italy

## Abstract

**Background:**

Healthcare-associated infections (HAIs) represent a serious burden to individual safety and healthcare sustainability. Identifying which patients, procedures and settings are most at risk would offer a significant contribution to HAI management and prevention. The purpose of this study is to estimate 1) orthopaedic implantable device-related infection (OIDRI) prevalence in Italian hospitals and 2) the gap between the remuneration paid by the Italian healthcare system and the real costs sustained by Italian hospitals to treat these episodes.

**Methods:**

This is a cross-sectional study based on hospital discharge forms registered in 2012 and 2014. To address the first goal of this study, the national database was investigated to identify 1) surgical procedures associated with orthopaedic device implantation and 2) among them, which patient characteristics (age, sex), type of admission, and type of discharge were associated with a primary diagnosis of infection. To address the second goal, 1) each episode of infection was multiplied by the remuneration paid by the Italian healthcare system to the hospitals, based on the diagnosis-related group (DRG) system, and 2) the total days of hospitalization required to treat the same episodes were multiplied by the average daily cost of hospitalization, according to estimates from the Ministry of the Economy and Finance (MEF).

**Results:**

In 2014, 1.55% of the total hospitalizations for orthopaedic device implantation procedures were associated with a main diagnosis of infection, with a negligible increase of 0.04% compared with 2012. Hip and knee replacement revisions, male patients and patients older than 65 years were more exposed to infection. A total of 51.63% of patients were planned admissions to the hospital, 68.75% had an ordinary discharge to home, and 0.9% died.

The remuneration paid by the healthcare system to the hospitals was € 37,519,084 in 2014, with 3 DRGs covering 70.6% of the total. The cost of the actual days of hospitalization to treat these episodes was 17.5 million more than the remuneration received.

**Conclusions:**

The OIDRI prevalence was lower than that described in recent surveys in acute care settings, although the numbers were likely underestimated. The cost of treatment varied significantly depending on the remuneration system adopted.

## Background

Healthcare-associated infections (HAIs) represent a serious burden to individual safety and healthcare sustainability on a global scale [1, 2]. In Europe, 3,2 million patients are estimated to receive an HAI diagnosis each year, of whom approximately 37,000 die [3]. Point prevalence surveys from 23 European countries estimated HAI prevalence to be 6.5% in acute care hospitals and 3.9% in long-term care facilities [4]. This is consistent with recent trends observed in Italian healthcare settings, where the prevalence of HAI episodes was estimated to be between 6.5 and 7.1% in acute care [5, 6] and between 3.4 and 3.9% in long-term care settings [6, 7]. The levels of antimicrobial resistance were also high in both settings, most frequently affecting the bloodstream, lower respiratory tract, and surgical site [5, 8–11].

Although estimating HAI incidence, complications, and attributable mortality is challenging [12], evidence suggests that systematic surveillance can help to prevent and reduce their burden [13–15]. These data highlight the need to identify which patients, procedures, settings and devices are most at risk.

Surgical procedures requiring the use of prosthetic devices are particularly exposed to the risk of infection, and among these, orthopaedics raise particular concerns [16–20]. Many international studies have investigated which variables are associated with the onset of infections in orthopaedic implantation surgery, identifying the most common microorganisms, procedures, prostheses, patients, physical conditions and comorbidities involved, from severe obesity to cancer and chronic HIV infection [21–23]. Prospective observational studies with the same goal have been recently published both in elective surgery and traumatology. Amlie et al. [16] investigated whether selected demographic characteristics (age and sex), clinical classifications (body mass index: BMI, and American Society of Anaesthesiologists: ASA), surgery duration, length of (hospital) stay (LOS), type of prosthesis (cemented versus uncemented), and healthcare pathways (fast-track or not) were associated with a higher risk of revision surgery due to deep infection following total hip arthroplasty, evaluating 4406 patients up to 3 months after surgery, and consequently finding both negative and positive significant correlations. Kumar et al. [18] investigated which type of microorganisms caused early postoperative wound infection among 80 patients who underwent implant surgery for close fracture treatment, finding *Staphylococcus aureus* (SA) as the major cause of infection (39%), followed by *Klebsiella* spp. (17%) and *Pseudomonas* spp. (15%), as well as which type of antibiotics they were most sensitive to, in order to support appropriate pharmacological care and prevent the emergence of more resistant strains of pathogens. This is consistent with findings from a previous retrospective observational study based on a more limited cohort of patients affected by chronic post-traumatic osteomyelitis and infected nonunion of the tibia, in which SA was found to be responsible for most of the postoperative wound infections, underlining the need for a safer approach to perioperative care [24]. Further studies have been recommended to improve infection prevention by means of identifying risky behaviours and conditions and improve infection treatment by translating innovative strategies from research to clinical practice [16, 19].

The Italian healthcare service (Servizio Sanitario Nazionale, SSN) relies on routinely implemented databases that offer useful information to improve the safety of medical devices and prosthetic surgery [25, 26]. However, no epidemiological or economic estimation of the impact of orthopaedic implantable device-related infections (OIDRIs) has yet been performed on a national scale.

Considering the public and private interest in reducing OIDRIs, in 2016, the Italian Ministry of Health (which represents the main healthcare funder and regulator) and the National Agency for Regional Health Services (Agenas, which represents the institutional link between the central, regional and local levels of government) agreed with the Confindustria Dispositivi Medici (which represents the healthcare industry) to set up a study protocol to estimate the impact of OIDRIs based on data from all national hospitals [27].

The aim of this study is to estimate 1) OIDRI prevalence in Italian hospitals, both in general and in relation to specific procedures, demographic characteristics of patients, type of admission and type of discharge, and 2) the gap between the remuneration paid by the SSN to Italian hospitals and the real costs sustained by the latter to treat OIDRI episodes.

## Methods

### Study design

This is a retrospective cross-sectional study. Data collection, analysis and interpretation regarding OIDRI prevalence and costs in Italian hospitals were reported by following the items of the STROBE checklist for cross-sectional studies, although the order may vary to improve the clarity of the manuscript [28].

### Data source and collection

The estimations were performed by screening all hospital discharge forms (HDFs) registered in 2012 and 2014. Established in 1991, the HDF database is the primary source of information for clinical practice in and remuneration of Italian hospitals, based on the ICD-9-CM diagnosis and procedure codes. Each form includes up to six codes organized hierarchically, among which the first diagnosis code and the first procedure code represent the main cause of hospitalization and the main treatment performed to cure a certain disease, respectively, and hence the associated standard remuneration. The form also includes information on LOS and type of admission (i.e., planned or urgent), type of discharge (i.e., home, another hospital, rehabilitation unit, nursing home, death) and demographic characteristics of the patient (age, sex and region of origin). HDFs are registered to the national database regardless of the nature of the hospital that provided the treatment (i.e., public or private-contracted), offering a comprehensive sample of HAI-related hospitalizations.

### Epidemiological analysis

To define a cross-sectional representation of OIDRIs given the information available in the HDFs, the researchers 1) took into consideration only acute care admissions (no rehabilitation or long-term care), since OIDRIs are treated within this setting, and 2) relied on a focus group composed of clinical, scientific and technological experts, who identified a list of diagnostic and procedural codes significantly associated with orthopaedic prosthesis implantation. As a result, 2 diagnostic and 142 procedure codes from the ICD-9-CM were clustered into 15 consistent macro categories. An additional 16th category was created to group 49 additional medical procedures non-specifically associated with orthopaedic diagnosis and treatment (“general procedures associated”: diagnostic exams, drug injections, excisional wound infection or burn debridement) (see Additional file 1).

The authors selected only those forms that reported an orthopaedic implantation procedure and a diagnosis of infection as primary codes [29, 30]. This decision was made for several reasons: 1) the degree of HDF compilation varies significantly among professionals, since some operators report only the main reason for admission, while others fill the form more comprehensively; therefore, selecting only primary codes is a way to ensure that the patient was admitted due to an episode of OIDRI; 2) a diagnosis of infection may be reported in the form of a secondary (or further) admission even if the actual episode of infection occurred and was treated in a previous admission; therefore, a singular episode of infection followed by complications and readmissions could be reported two or more times.

The HDF database was analysed for 2 years, 2012 and 2014, to measure the eventual change in overall prevalence. Detailed information on which procedures, patient ages and sex, types of admission and types of discharge were more frequently associated with an episode of OIDRI are reported only for 2014 in order to describe the latest data available and offer a more concise view of the results.

All patients admitted to Italian hospitals for acute care in 2012 and 2014 with a primary procedure of orthopaedic device implantation and a primary diagnosis of infection reported in the HDF were included in the study. Since each HDF includes information about the region of origin of the patient but no information about the region of the hospital where the infection occurred (which would be relevant for the sake of policy and prevention), this demographic characteristic was not considered.

Screening the database for primary procedure codes resulted in a sample of 348,178 (2012) and 336,593 (2014) admissions; among them, subgroups of 5266 (2012) and 5214 (2014) episodes reporting an infection as the primary diagnosis were selected. Among the episodes registered in 2014, it was possible to identify which procedures, ages, sex, types of admission and types of discharge were most frequently associated with an episode of OIDRI, allowing the authors to answer the first question.

### Economic analysis

To answer the second question, the economic impact of OIDRIs was calculated by multiplying 1) each episode of infection by the cost of the corresponding standard remuneration, based on the diagnosis-related group (DRG) system, and 2) the total days of hospitalization required to treat these episodes in 1 year by the average daily cost of hospitalization, as estimated by the Ministry of Economics and Finance [31], and adjusted for inflation to 2014 values. The adoption of both economic estimations allowed the authors to investigate the gap between the standard costs expected by the Ministry of Health to treat an OIDRI and the real costs incurred by Italian hospitals.

### Statistical analysis

All statistical analyses were performed using R software version 3.6.2 (R Foundation for Statistical Computing, Vienna, Austria). The Kolmogorov-Smirnov test was applied to test the normal distribution of data. Tests for equality of proportions were performed to assess infections in different categories of age, sex, type of admission and type of intervention. Two-way analysis of variance was applied to evaluate the influence of the type of intervention, infection and their interaction on the LOS. Then, a multilevel mixed-effects linear model was developed using the R package lme4 [32] to estimate the increased LOS for patients affected by an infection, adjusted for the type of intervention. Descriptive statistics were obtained for each parameter. Categorical variables are reported as counts and absolute or cumulative percentages. For continuous numerical variables, the means, standard deviations (STDs) or medians and interquartile ranges (IQRs) are reported. A *p*-value < 0.05 was considered statistically significant.

**Fig. 1:**
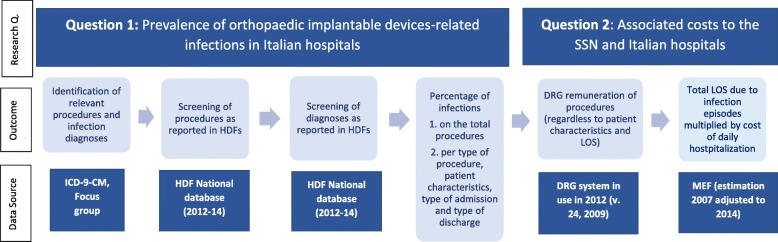
Methodological overview. ICD-9-CM: International Classification of Diseases - 9th revision - Clinical Modification. HDF(s): Hospital discharge form(s). SSN: Servizio Sanitario Nazionale. DRG(s): Diagnosis-related group(s). LOS: Length of Stay. Research Q.: Research Question

Before performing the analyses, the heterogeneity of the HDF database was addressed by using tools available in STATA software (version 16) to 1. uniform details on the region, local healthcare unit and hospital; 2. uniform major diagnoses and the associated DRGs; 3. verify appropriate code reporting based on ICD-9-CM diagnoses and procedures; 4. identify missing data; and 5. integrate DRG tariffs in order to uniform the analysis sources. Figure 1 provides an overview of the research methodology.

## Results

### OIDRI prevalence in Italian hospitals

Screening the HDF database for acute care hospitalization and primary codes of orthopaedic implantation devices, 348,178 procedures were performed in Italian hospitals in 2012 and 336,593 in 2014. Among them, 5266 hospitalizations reported an infection as the primary diagnosis in 2012 and 5214 in 2014, with a prevalence of 1.51 and 1.55%, respectively. The negligible increase in prevalence reported over the 2 years (0.04%) confirmed the decision to only report detailed results for 2014.

Screening the database by type of procedure showed that general procedures associated with orthopaedic diagnosis and treatment (71.92%), generic musculoskeletal device implantation (63.46%), and generic musculoskeletal device removal (39.88%) had the highest prevalence in OIDRI episodes (Table 1). General procedures associated with orthopaedic diagnosis and treatment (n. 1895), hip replacement revision (n. 1203) and knee replacement revision (n. 739) had the highest absolute number of episodes of infection. These differences were statistically significant (*p* < 0.0001).

**Table 1:** Prevalence of infections per type of procedure (2014)

Macro category	Total	With infection	Prevalence (%)
1. Primary hip replacement	89,242	37	0.04%
2. Hip replacement revision	7292	1203	16.50%
3. Other hip procedures	1000	0	0.00%
4. Primary knee replacement	61,923	42	0.07%
5. Knee replacement revision	3017	739	24.49%
6. Other knee procedures	115	0	0.00%
7. Lower limb implantations (femur, tibia, feet)	99,189	20	0.02%
8. Lower limb revisions (femur, tibia, feet)	27,492	448	1.63%
9. Other lower limb procedures	167	0	0.00%
10. Higher limb implantations (shoulder, arm, hand)	7900	18	0.23%
11. Higher limb revisions (shoulder, arm, hand)	12,540	138	1.10%
12. Other higher limb procedures	13,313	7	0.05%
13. Generic musculoskeletal implantation	156	99	63.46%
14. Generic musculoskeletal removal	163	65	39.88%
15. Other orthopaedic procedures	10,449	503	4.81%
16. General procedures associated	2635	1895	71.92%
Total	**336,593**	**5214**	**1.55%**

Age had a significant influence on the rate of infection, demonstrating an increasing trend (Table 2, *p* = 0.0002). Indeed, 30.36% of patients facing infection (n. 1583) were older than 75 years old, while 32.30% (n. 1684) were between 65 and 75 years old. In contrast, low rates of infection were observed in paediatric patients, with just 5 patients younger than 5 years old and 72 patients between 5 and 15 years old.

**Table 2:** Prevalence of infections, per age (2014)

Age	Total	with infection	%
00–05	1347	5	0.37%
05–15	9378	72	0.76%
16–25	11,550	162	1.38%
26–35	11,435	137	1.18%
36–45	18,818	271	1.42%
46–55	29,884	441	1.45%
56–65	48,676	859	1.74%
66–75	83,552	1684	1.98%
> 75	121,953	1583	1.28%
Total	336,593	5214	1.55%

Screening the database by sex, 38.9% of patients (n. 130,215) were male and 61.1% were female (n. 206,378). Males accounted for 45.5% of OIDRI episodes, with a significantly higher risk than females (risk ratio: 1.31, 95% CI: 1.26–1.36; *p* < 0.0001).

Screening the database by type of admission, 51.63% of patients with infections (n. 2692) had planned admission to the hospital, 23.86% (n. 1244) were admitted due to an emergency, while information about the remaining 24.52% (1408) was not available. These values significantly differed from those of patients without infection, for whom emergency admissions were more frequent (42.1%, *p* < 0.0001).

Screening the database by type of discharge, 68.75% of patients were discharged to home without an infection, followed by patients transferred to rehabilitation facilities (12.91%) and to other wards in the same hospital (11.4%). Substantial differences emerged between patients with and without infection, as reported in Table 3. In particular, patients with infection were more frequently discharged to home or transferred to other hospitals, while in the absence of infection, higher rates of transfers to rehabilitation facilities, other wards in the same hospital or nursing homes were observed.

**Table 3:** Type of discharge in patients with and without infection (2014)

Type of discharge	With infection		Without infection		p-value
	N	%	n	%	
Dead	47	0.90%	2619	0.79%	0.413
Ordinary discharge to home	3932	75.41%	227,822	68.75%	< 0.0001
Ordinary discharge to nursing home	80	1.53%	8926	2.69%	< 0.0001
Discharge to assisted home care	11	0.21%	581	0.18%	0.658
Voluntary discharge	52	1.00%	1215	0.37%	< 0.0001
Transfer to other hospitals	163	3.13%	6835	2.06%	< 0.0001
Transfer to other wards in the same hospital	531	10.18%	37,788	11.40%	0.0064
Transfer to rehabilitation facilities	351	6.73%	42,782	12.91%	< 0.0001
Ordinary discharge to assisted home care	47	0.90%	2809	0.85%	0.7311
**Total**	**5214**	**100%**	**331,377**	**100%**	**–**

### OIDRI costs to the Italian healthcare system

The 5214 OIDRI hospitalizations registered in 2014 were classified to 36 different DRGs, representative of both day hospital and ordinary admissions. Multiplying each episode of infection by the cost of the standard DRG remuneration, the reimbursement provided by the SSN to Italian hospitals was € 37,519,084 (standard costs).

The first three DRGs (Hip or knee replacement, revision; Rehabilitation assistance for diseases of the musculoskeletal system and connective tissue; Local excision and removal of internal fixation means except hip and femur without complications and comorbidities) covered 70.6% of the total expenditure. The total sum of the first 10 DRGs covered 94% of the total expenditure, among which 9 were surgical procedures and 1 was medical (Table 4).

**Table 4:** 10 Most impacting DRGs on the total expenditure (2014)

DRG cod.	DRG description	Procedure	N.	%	Cumulative %
545	Hip or knee replacement, revision	S (Surgical)	1661	31.9	31.9
249	Rehabilitation assistance for diseases of the musculoskeletal system and connective tissue	M (medical)	1057	20.3	52.1
538	Local excision and removal of internal fixation means except hip and femur without Complications and comorbidities (CC)	S	965	18.0	70.6
217	Wound debridement and cutaneous transplant except hand, for diseases of the musculoskeletal system and connective tissue	S	342	6.6	77.2
211	Hip and femur surgery, except for major joints, age > 17 without CC	S	217	4.2	81.4
210	Hip and femur surgery, except for major joints, age > 17 with CC	S	179	3.4	84.8
230	Local excision and removal of intramedullary pinning of hip and femur	S	156	3.0	87.8
537	Local excision and removal of intramedullary pinning except hip and femur with CC	S	152	2.9	90.7
544	Major joints replacement or lower limbs reimplantation	S	90	1.7	92.4
502	Knee surgery reporting infection as a major diagnosis without CC	S	82	1.6	94.0

The total LOS reported in Italian hospitals for treating an OIDRI episode in acute care was 72,091 days in 2014. Multiplying this value by the daily cost of hospitalization estimated by the Ministry of Economics and Finance (€ 762.97), the total expenditure incurred by Italian hospitals was € 55.0 million (real costs).

LOS was significantly influenced by both the type of procedure (*p* < 0.0001) and the presence of infection (*p* < 0.0001) (Table 5). Given the interaction of these two variables, a multilevel linear model was developed taking into account the effect of the different interventions. The model revealed that once adjusted for the influence of the different categories, LOS was 3.46 days longer in cases of infection than in standard procedures. Considering the procedures with the highest impacts on the infection rate, i.e., hip and knee arthroplasty revision surgeries and general musculoskeletal implantation or removal (categories n. 2, 5, 13 and 14, Table 5), the great variability in LOS within and between groups emerged clearly. The LOS for hip and knee revision surgeries ranged between 1–230 and 1–397 days, respectively. Similarly, the extreme values for LOS in general implantation or removal were 1 and 217 days. Nevertheless, while it is difficult to identify a pattern in these heterogeneous groups for LOS in patients with and without infection, due to the close medians and the largely overlapping IQR, an increase in LOS for patients undergoing hip or knee arthroplasty revisions is evident. A difference of 3 days between the median LOS of patients with and without infection confirms the findings of the statistical model in both categories. Smaller IQR overlapping was also observed, especially for knee arthroplasty revisions (6–11 vs 8–18).

**Table 5:** Variations in LOS due to an infection (2014)

	Hospitalization without infection				Hospitalization with infection			
Procedure category	N.	Total LOS	Median	IQR	N.	Total LOS	Median	IQR
Primary hip replacement	89,205	895,623	9.00	7.00–12.00	37	818	14.00	12.00–25.00
Hip replacement revision	6089	79,228	10.00	7.00–15.00	1203	21,643	13.00	8.00–22.00
Other hip procedures	1000	8642	7.00	4.00–11.00	0			
Primary knee replacement	61,881	478,994	7.00	5.00–9.00	42	598	12.00	8.25–17.00
Knee replacement revision	2278	22,583	8.00	6.00–11.00	739	11,317	11.00	8.00–18.00
Other knee procedures	115	1208	8.00	5.00–12.00	0			
Lower limb implantations (femur, tibia, feet)	99,169	1,054,608	9.00	6.00–13.00	20	412	15.00	8.75–20.25
Lower limb revisions (femur, tibia, feet)	27,044	74,678	1.00	1.00–3.00	448	4025	3.00	1.00–9.00
Other lower limb procedures	167	698	3.00	1.00–5.00	0			
Higher limb implantations (shoulder, arm, hand)	7882	61,800	6.00	4.00–10.00	18	140	7.00	3.25–10.00
Higher limb revisions (shoulder, arm, hand)	12,402	24,501	1.00	1.00–2.00	138	644	2.00	1.00–5.00
Other higher limb procedures	13,306	72,733	3.00	2.00–6.00	7	59	5.00	1.50–10.00
Generic musculoskeletal implantation	57	1184	11.00	6.00–24.00	99	1293	10.00	7.00–17.00
Generic musculoskeletal removal	98	1446	9.00	5.25–15	65	899	11.00	8.00–18.00
Other orthopaedic procedures	9946	34,537	1.00	1.00–3.00	503	3821	5.00	1.00–10.00
General procedures associated	740	13,181	12.00	6.00–22.00	1895	26,602	9.00	3.00–19.00
**Total**	**331,379**	**2,825,644**	**7.00**	**4.00–11.00**	**5214**	**72,091**	**9.00**	**5.00–18.00**

*LOS* Length of Stay.

*IQR* Interquartile Range.

## Discussion

The prevalence of OIDRIs in the analysed database was lower than the European HAI prevalence, Italian HAI prevalence and Italian OIDRI prevalence, as described in recent surveys in acute care settings [4–7]. The decision to include only primary procedure and diagnosis codes may explain this underestimation, just as - in contrast - the inclusion of all episodes without checking for false positives such as multiple reports would probably result in an overestimation. Checking the relevance of each singular case is more suitable for a local survey (i.e., single-hospital or regional network) than for a national survey, as the absence of uniform national guidelines may lose some relevant amount of HDF information to poor compliance. Indeed, surveys are increasingly being performed in Italian regions and university hospitals [1, 6, 33], while systematic surveillance networks are being tested on an international scale [13]. Two reviews and one retrospective observational study found OIDRI treatment success rates to vary between 57 and 88% [34–36]. Although this study was unable to detect the rates of successful treatment, the 68.75% of patients discharged to home without an infection is aligned with this finding. Less than 1% died. A total of 30.09% of patients were discharged or transferred to other wards or facilities, out of whom 22.68% had an infection, which underlines the need to maintain surveillance over the full cycle of postoperative care [37]. Due to the anonymity of the information contained in an HDF, this study could not identify patients who had repeated hospitalizations or track their movement through different healthcare wards, facilities and providers.

A review of orthopaedic and trauma-related infections found that the rate varied significantly depending on the type of surgery (procedure) and the type of admission (planned vs. urgent), introducing a list of current and developing interventions to improve prevention and treatment [19]. A prospective observational study on a large population found an increased risk of revision surgery due to deep infection following hip arthroplasty in male patients and accelerated pathways, although the combination of local infiltration analgesia, drain cessation and early mobilization did not allow us to identify the reason more precisely; in contrast, BMI, physical status of the patient, type of prosthesis (cemented vs. uncemented, different labels), type of surgery and LOS were not associated with an increase in revision risk [16].

The international breadth of these studies does not allow a systematic comparison with the results of this study, considering the specificity of the methodology and database employed here to describe OIDRI prevalence in Italian hospitals. However, correlations between OIDRI frequency, type of procedure and type of admission were confirmed in the present analysis. General procedures associated with orthopaedics diagnosis and treatment, musculoskeletal device removal and musculoskeletal device implantation had the highest prevalence within the sample, while general procedures associated with orthopaedic diagnosis and treatment, hip replacement revision and knee replacement revision had the highest numbers in absolute terms. This is consistent with studies reporting prosthetic joint infection as a recurrent complication of joint replacement procedures, which may reach up to 20% in cases of revision [38, 39]. The rate is roughly confirmed by this research, as knee and hip replacement revisions were associated with an infection in 16.50 and 24.49% of patients, respectively. A multicentre randomized prospective study on 380 patients from six European orthopaedic centres found that the use of a fast-resorbable, antibiotic-loaded hydrogel implant coating reduced the rate of surgical site infection after hip or knee joint replacement, either with a cementless or a hybrid implant [40].

A systematic review and meta-analysis on patient-related risk factors for periprosthetic joint infection after total joint arthroplasty [41] found that male patients were more exposed to this type of infection, which is supported by the present study and by a large prospective study performed on fast-track total hip arthroplasty [16], but found no correlations between age and exposure to OIDRI. In contrast, this study recommends caution when treating older patients, consistent with other studies performed in Italian hospitals, which found an age of 65 years or more to be a risk factor for increased HAIs and orthopaedic surgical site infections [1, 20].

With the exception of age and sex, many patient characteristics (smoking, alcohol consumption, BMI, drug consumption, mental health and chronic disease) are modifiable before surgery and could potentially be employed to identify patients at high risk of developing specific types of infections to target appropriate interventions [41]. This advice is particularly relevant, considering that nearly half of the patients affected by OIDRI had elective surgery or planned hospitalization, which offers significant room for evaluating and preventing patient risk [19, 42–44]. Increasing interest is also being given to improve the treatment of vulnerable patients affected by specific comorbidities such as chronic HIV infection and cancer. A prospective comparative study found that the combination of prolonged prophylactic cefuroxime therapy and systematic antiretroviral therapy reduced postoperative infection rates to similar levels as those of non-HIV carriers [23]. A retrospective comparative study on orthopaedic oncology patients affected by deep infections after endoprosthetic replacement found SA and coagulase-negative *staphylococci* to be the most common microorganisms responsible for the infection; since most of these microorganisms demonstrated resistance to methicillin antibiotics, they represent a serious further complication in the management of these patients [22]. Diabetes, malnutrition, use of tobacco, obesity, nasal SA incubation, preoperative and postoperative anaemia, and urinary tract infections also increase the risk of bone infection after orthopaedic implantation surgery in traumatology; a retrospective observational study on twenty patients affected by bone infection after orthopaedic implantation surgery described an equal number of combinations between type of fracture, velocity of trauma (medium or low), procedure performed, personal history (smoking and/or alcohol drinking), onset of infection (early or late), causative agent, sensitivity to different antibiotics, and surgical result (nonunion, osteomyelitis, implant failure). The specific surgeon performing the operation, the length of wound exposure to air and a previous improper use of antibiotics were also shown to play a significant role. Although performed on a small number of patients, the study confirmed SA to be responsible for most of the infections (60%); furthermore, although SA is sensitive to antibiotics such as linezolid, clindamycin and vancomycin, the study calls for new approaches to improve the safety of perioperative care, which may be worth investigating on a larger scale [24]. Indeed, judicial use of antibiotics in the treatment of orthopaedic surgical site infections is a fundamental requirement for preventing the emergence of more resistant strains of pathogens [18].

The epidemiological and economic focus of the present research does not include a detailed review of the current efforts to improve the prevention, diagnosis and treatment of infections. However, contributions from international guidelines, reviews and conference proceedings are frequent in the literature. Among them are the multidisciplinary “Consensus document for the diagnosis of prosthetic joint infections”, endorsed by the European Society of Clinical Microbiology and Infectious Diseases, and elaborated consistently with the Oxford Centre for Evidence-based Medicine (OCEBM) by the European Association of Nuclear Medicine (EANM), the European Bone and Joint Infection Society (EBJIS), and the European Society of Radiology (ESR) [45]; the Second International Consensus Meeting (ICM) on musculoskeletal infection, which reports specific information about the prevention, diagnosis and treatment of infections of the foot and ankle, hip and knee, shoulder and elbow, and spine, and includes disciplines such as oncology, paediatrics, sports and trauma orthopaedic surgery [46]; the American Academy of Hip and Knee Surgeons approach to the diagnosis of periprosthetic joint infection, based on erythrocyte sedimentation rate, C-reactive protein, eventual selective aspiration of the joint, and synovial fluid white blood cell count [47]; a systematic review with meta-analysis of the effectiveness of debridement, antibiotics and implant retention for the treatment of prosthetic joint infections, which was shown to be particularly successful against acute postoperative infections and infections of the hip and shoulder joints [41], but not in a small cohort of traumatological patients [24]; and an overview of the treatment and prevention of prosthetic joint infections based on consensus documents published on the topic before 2014, including information on epidemiology, economic impact, risk factors, clinical manifestations, pathogenesis, the role of biofilm and propagation of infection [48]. Ionic silver, active and passive immunization for SA, antimicrobial peptides and immunomodulatory peptides, quorum sensing inhibitors and biofilm degrading enzymes were evaluated by an international review both in terms of research status and current clinical application to support the translation of knowledge from clinical trials to future routine practice (19).

It was more difficult to describe preventive measures to avoid infection following general procedures associated with orthopaedics diagnosis and treatment because different procedures require different specific arrangements. For this purpose, an index of international studies, managed by the Italian Association of Clinical Microbiologists (Associazione Microbiologi Clinici Italiani, AMCLI), may regularly be consulted by searching for specific keywords [49].

Although highly representative of the number of admissions registered in Italian hospitals within a certain interval of time, the HDF database between 2012 and 2014 excluded information on patient comorbidities, anamnesis and history of hospitalization, which were introduced by later legislation [50]. Therefore, the database does not represent the entire epidemiological information potentially available in the country, such as specific correlations between patient characteristics, type of infections, procedures and devices. In turn, the ICD system still adopted by the Ministry of Health (ICD-9-CM, year 2007) does not represent the entire clinical and technological know-how currently available in Italian hospitals, which is also reflected in the gap between standard remuneration and real costs.

Italian hospitals are funded by the SSN through the DRG remuneration system. The overall reimbursement paid by the SSN to Italian hospitals to treat OIDRIs was approximately € 37.5 million in 2014, of which 70% could be prevented by improvement efforts on 3 DRGs only (Hip or knee replacement, revision; Rehabilitation assistance for diseases of the musculoskeletal system and connective tissue; Local excision and removal of internal fixation means except hip and femur without complications and comorbidities).

However, the total expenditure sustained by Italian hospitals to treat OIDRIs in the same year was € 55.0 million, resulting from the difference between the expected LOS and real LOS, which is not adequately captured by the current DRGs. The latest version of the DRG system adopted in Italy (version 24, year 2009) is based on an outdated version of the ICD system, which does not allow precisely distinguishing between hospitalizations and procedures aimed at treating an infection, complicated by an infection, and free from infection. Furthermore, these classifications are unable to identify the type and number of comorbidities that can affect the increasing numbers of elderly patients suffering from one or more chronic diseases, which would be a fundamental result for providing patient-oriented care. General indicators of comorbidity (such as CC, “con complicanze”) do exist, but no more specific information is taken into account, which prevents remunerations from being adapted to the real costs sustained by the hospital to treat different patients. As a result, although OIDRI episodes required on average 3.46 more days of admission than what was expected by standard implantation procedures, both OIDRI- and non-OIDRI-based hospitalizations were remunerated under the same cost. The difference between standard costs and real costs, which the present study estimated at € 17.5 million, underlines how the DRG system now adopted by the SSN does not represent the actual expenditure sustained by Italian hospitals to treat OIDRIs. The obsolete classification systems employed by the SSN and described here represent a limitation of the present study. At the same time, this study provides evidence in support of the need to update and integrate these systems.

## Conclusions

The current DRG system needs to be updated to 1) provide hospitals with proper funding based on effective clinical procedures, human efforts, available technologies, and current resource consumption and 2) estimate more clearly the subsequent epidemiological and economic burden sustained by Italian hospitals and the national healthcare system [51]. In addition, uniform HDF reporting must be supported by validated national guidelines to reduce the variety in compliance.

Given the high socioeconomic impact of musculoskeletal infections [52], of which prosthetic joint infections are of particular concern [33], reducing device-related preventable infections is a key element for delivering value-based healthcare in terms of personal value (improved safety and patient-centred care), technical value (improved quality of care and device purchasing), allocative value (increased cost-effectiveness and reduced readmissions in the long term) and social value (reduced human and economic burden to society) [53, 54].

In a phase of financial constraints, it is fundamental to employ the limited resources available to the healthcare system to fund those technologies and procedures that provide the best value. Updating the current classification systems is an important but sometimes neglected task, which could foster quality and efficiency of care well beyond the field of orthopaedics. All countries characterized by similar epidemiological, demographic, institutional and financial challenges could take advantage of the information provided.

## Supplementary information

**Additional file 1.** Orthopaedic implantation procedures and infection diagnoses identified by the focus group, based on ICD-9-CM (International Classification of Diseases - 9th revision - Clinical Modification). The table contains the ICD-9-CM codes of procedure and diagnosis which the focus group has attributed to orthopaedic implantation device-related infections (see Methods section).

## Data Availability

All data generated or analysed during this study are included in this published article.

## Abbreviations

AGENAS: Agenzia Nazionale per i Servizi sanitari regionali
DRG(s): Diagnosis-related group(s)
HAI(s): Healthcare-associated infection(s)
HDF(s): Hospital discharge form(s)
ICD-9-CM: International Classification of Diseases - 9th revision - Clinical Modification
LOS: Length of stay
OIDRI(s): Orthopaedic implantation device-related infection(s)
SSN: Servizio Sanitario Nazionale
