# Supplementary material for: Prevalence and burden of orthopaedic implantable-device infections in Italy: a hospital-based national study
Source: BMC Infect Dis. 2020 May 12;20:337. doi: 10.1186/s12879-020-05065-9 (PMC7216513; doi:10.1186/s12879-020-05065-9)
Supplement: Supplementary file 1 — Additional file 1. Orthopaedic implantation procedures and infection diagnoses identified by the focus group, based on ICD-9-CM (International Classification of Diseases - 9th revision - Clinical Modification). The table contains the ICD-9-CM codes of procedure and diagnosis which the focus group has attributed to orthopaedic implantation device-related infections (see Methods section). [file 12879_2020_5065_MOESM1_ESM.docx]

| Additional file 1. Orthopaedic implantation procedures and infection diagnoses identified by the focus group, based on ICD-9-CM (International Classification of Diseases - 9th Revision – Clinical Modification) | |
| --- | --- |
| Diagnoses | |
| Code | Description |
| 996.66 | Infection and inflammatory reaction due to internal joint prostheses |
| 996.67 | Infection and inflammatory reaction due to other internal orthopaedic device, implant, and graft. |
| Procedures | |
| Macro category | Codes |
| 1. Primary hip replacement | 81.51, 81.52 |
| 2. Hip replacement revision | 00.70, 00.71, 00.72, 00.73, 80.05, 81.53 |
| 3. Other hip procedures | 91.33, 70.81, 87.64, 68.06, 94.65, 79.26, 95.01, 83.67, 75.22, 96.82, 91.40, 88.08 |
| 4. Primary knee replacement | 81.54 |
| 5. Knee replacement revision | 80.06, 81.55, 00.81, 00.82, 00.83, 00.84 |
| 6. Other knee procedures | 54.41, 47.47, 83.45, 94.94, 66.84, 58.24, 73.70, 74.13, 79.55 |
| 7. Lower limb implantations (femur, tibia, foot) | 81.56, 78.15, 78.55, 79.15, 79.35, 78.17, 78.57, 79.16, 79.36 |
| 8. Lower limb revisions (femur, tibia, foot) | 78.65, 78.67, 78.68, 80.08, 80-07 |
| 9. Other lower limb procedures | 86.78, 93.28, 86.92, 93.35, 93.42, 50.00, 82.80, 70.30, 87.36, 94.00, 94.15, 94.44, 52.31, 94.87, 95.16, 95.23, 95.45, 95.74, 96.75, 60.04, 95.81 |
| 10. Higher limb implantations (shoulder, arm, hand) | 81.81, 78.12, 78.52, 79.11, 79.31, 81.73, 81.84, 81.80, 57.30, 51.23 |
| 11. Higher limb revisions (shoulder, arm, hand) | 78.61, 78.62, 78.63, 80.02, 80.03, 80.04, 93.64, 93.71, 93.79, 93.86, 93.93, 94.08, 94.22, 94.29, 94.36, 73.27, 94.51, 94.58, 87.93, 95.09, 95.66, 95.95, 61.71, 96.17, 96.89, 93.57 |
| 12. Other higher limb procedures | 93.64, 93.71, 93.79, 93.86, 93.93, 94.08, 94.22, 94.29, 94.36, 73.27, 94.51, 94.58, 87.93, 95.09, 95.66, 95.95, 61.71, 96.17, 96.24, 96.89, 93.57 |
| 13. Generic musculoskeletal implantation | 84.56 |
| 14. Generic musculoskeletal removal | 84.57 |
| 15. Other orthopaedic procedures | 81.92, 76.97, 78.69, 81.91, 91.91, 91.98, 92.05, 92.12, 92.20, 92.99, 93.06, 93.14, 35.04, 93.21, 82.59, 78.40, 87.50, 83.24, 38.51, 75.94, 96.03, 88.22, 96.10, 96.97, 83.02, 56.36, 40.82, 78.68, 99.78, 82.15, 85.84, 00.80 |
| 16. General procedures associated to orthopaedic diagnosis and treatment | 90.59, 93.01, 93.11, 90.52, 90.53, 91.32, 91.51, 91.52, 91.53, 91.54, 91.59, 91.62, 91.63, 91.72, 91.73, 91.79, 91.82, 91.83, 91.93, 92.05, 92.14, 92.16, 92.18, 92.19 |
